# The prevalence of and survival in Mucopolysaccharidosis I: Hurler, Hurler-Scheie and Scheie syndromes in the UK

**DOI:** 10.1186/1750-1172-3-24

**Published:** 2008-09-16

**Authors:** David Moore, Martin J Connock, Ed Wraith, Christine Lavery

**Affiliations:** 1 Department of Public Health and Epidemiology, University of Birmingham, Birmingham, B15 2TT, UK; 2 Willink Biochemical Genetics Unit, Royal Manchester Children's Hospital, Hospital Road, Pendlebury, Manchester, M27 4HA, UK; 3 The Society for Mucopolysaccharidosis diseases (UK), Registered Charity No. 287034, MPS House, Repton Place, White Lion Road, Amersham, Buckinghamshire, HP7 9LP, UK

## Abstract

**Background:**

Mucopolysaccharidosis type I (MPS I) is a rare lysosomal storage disease subdivided into three phenotypes of increasing severity: Scheie, Hurler-Scheie and Hurler. To gauge the effectiveness of treatments and to determine the load likely to fall on health-care systems, it is necessary to understand the prevalence and natural progression of the disease especially with regard to life-expectancy. In general such data on the natural history of lysosomal storage diseases is sparse.

**Methods:**

Analysis of prevalence and patient survival in MPS I disease using a unique longitudinal data set initiated and maintained over a period of more than 20 years by the Society for Mucopolysaccharide Diseases (UK).

**Results:**

The birth prevalence of MPS I in England and Wales over the period 1981 to 2003 was 1.07/100,000 births and within ± 5% of estimates reported in several studies that examined reasonably large populations. The median survival for MPS I patients (including all phenotypes irrespective of various treatments) was found by Kaplan-Meier analysis to be 11.6 years. This result was driven by the relatively poor survival of patients with the Hurler phenotype who, irrespective of any treatments received, had a median survival of 8.7 years; when censoring for receipt of bone marrow transplant (BMT) was implemented median survival of Hurler patients was diminished to 6.8 years. The difference between these survival curves was statistically significant by log rank test and can be attributed to beneficial effects of BMT and or selection of patients with superior prognosis for intervention with BMT. Survival curves for Hurler patients who received and did not receive BMT were very different. Probability of survival at 2 year after BMT was ~68% and was similar to this after 5 years (66%) and ten years (64%); the mean age of Hurler patients at receipt of BMT was 1.33 years (range 0.1 to 3 years). Follow up was insufficient to determine median survival of the milder phenotypes however, unsurprisingly, this was clearly superior to that for Hurler patients.

**Conclusion:**

The birth prevalence of MPS I in England and Wales is 1.07/100,000 and the median survival for MPS I patients is 11.6 years.

## Background

Mucopolysaccharidosis type I (MPS I) is a panethnic, chronic and progressive, autosomal recessive lysosomal storage disease in which degradation of the glycoaminoglycans (GAGs) dermatan and heparan sulphate is deficient. First described by Hurler in 1919, a milder phenotype was later identified by Scheie in 1962 [1]. Currently three clinical syndromes are often referred to which, from severe through intermediate to mild phenotypes, are termed Hurler, Hurler-Scheie and Scheie. It is clear however that this is an oversimplification and that the full phenotypic spectrum of disease forms a continuum.

The deficient enzyme in MPS I is α-L-iduronidase responsible for removing terminal iduronic acid residues during the sequential degradation of dermatan and heparan sulphates [1]. The disease is characterised by inappropriate storage of these GAGs with accompanying organ enlargement, the excretion of abnormal quantities of GAGs in urine, and disrupted GAG turnover that especially affects connective tissue [1]. The multi-system sequelae result in clinical manifestations that vary between individuals but may include mental retardation, skeletal abnormalities, enlarged liver and spleen, respiratory problems, heart disease and reduced life expectancy [1].

Traditionally treatments for MPS I have aimed at relieving symptoms. More radical treatments have been explored including bone marrow transplantation which has become the treatment of choice for carefully selected Hurler patients. Enzyme replacement therapy (alpha-L-iduronidase, Aldurazyme^®^) is now available to treat the intermediate and milder phenotypes (Hurler-Scheie and Scheie) as well as recently more severely affected patients [2]. The development of enzyme enhancement therapy, substrate reduction strategies and also of gene therapy for lysosomal storage diseases and other metabolic disorders, and the various combinations of such strategies, are possible future developments.

In view of the extremely high cost of some new therapies that have been or are being developed under the exclusivity provided by orphan drug legislation it becomes important to estimate the health gains returned with use of these treatments so that a clear understanding can inform the deployment of limited health resources. Difficulties arise in taking appropriate account of the clinical and cost-effectiveness of such interventions because of the sparsity of good quality data as amply exemplified in recent Health Technology Assessment of enzyme replacement therapy for Gaucher's disease [3-5]. Many data-collecting initiatives are prompted by marketing of new technologies with or without recommendation from regulatory authorities. However such data collection will usually only gain good quality (i.e. prospective) information for patients on-treatment and fails to provide evidence about 'natural progression' of disease in the absence of the new technology since patients that are off-treatment are not representative of the whole disease population and are likely to differ from those on-treatment. Therefore there is very little evidence from such sources with which a new technology can be assessed. This leads to difficulties for physicians and health-care commissioners in determining the clinical and ultimately the relative cost-effectiveness of a technology under conditions of competing budgetary requests.

In order to gauge the effectiveness of new and future treatments it is necessary to understand the natural progression of the disease especially with regard to patients' quality of life and life-expectancy. In general such data on the natural history of lysosomal storage diseases is sparse. Here we analyse patient survival in MPS I and disease prevalence of MPS I using a unique longitudinal data set initiated and maintained over a period of more than 20 years by the Society for Mucopolysaccharide Diseases (UK). This data will be of interest to clinicians, health care authorities, commissioning bodies, those engaged in diagnosis of rare diseases and to patient societies and patient families.

## Methods

The Society for Mucopolysaccharide Diseases UK (SMD) made available anonomysed records of MPS I patients held in its registry. The Society has aimed to collect data on every UK MPS I sufferer. As most, if not all, patients are now seen at a small number of designated centres, most if not all UK patients are entered into the register. While the number of patients likely to be missing from the register is unknown, it is estimated by collators of the register and by clinicians treating MPS I patients to be zero or very few. Therefore, the dataset can be considered as virtually complete for all UK patients from 1981.

The dataset was analysed for patients entered in to the register between 1981 and May 2005. This registry holds information on birth dates and country of birth, diagnosed MPS I syndrome, receipt of bone marrow transplantation (BMT) and date of transplant, receipt of enzyme replacement therapy (ERT) and date of death. Date of diagnosis and genotype of a significant proportion of patients is also recorded but is incomplete; as would be expected [6] the recorded genotypes were highly variable and mostly private except for the W402X mutation which was fairly common.

### Prevalence

Due to the well-documented possible delays in diagnosis of MPS I, especially with regard to the milder syndromes, we considered births up to 2003 rather than more recent. Although the registry records are judged comprehensive, we considered that ascertainment for England and Wales was most likely to be complete and that these cases would also account for the vast majority of MPS I patients in the UK. The three-year running average by year for MPS I births was calculated and compared with the three-year running average for all births in England and Wales. These averages were utilised in order to smooth chance year on year fluctuations which might be expected to occur with rare events such as MPS I births. Total number of births in England and Wales was accessed from National statistics [7].

### Survival

Kaplan-Meier survival analysis, median survival estimates and log-rank tests were performed using Stats Direct and STATA (version 8) software for all patients in the registry.

## Results

At the time of analysis the registry held data on a total of 196 MPS I patients covering the 24 years 1981 to 2005. The patients were categorised according to syndrome as follows: 143 Hurler, 41 Hurler-Scheie, and 12 Scheie.

### Prevalence

**Figure 1:**
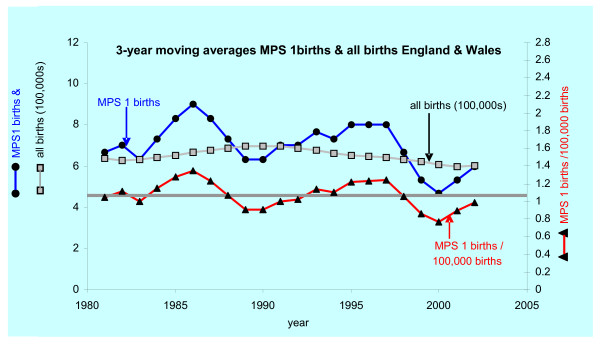
**Birth prevalence of MPS l**. The horizontal line represents the overall birth prevalence (1.07/100,000 live births) for MPS I in England and Wales.

Figure 1 shows the three-year running average for MPS I births and for all births in England Wales 1981 to 2003. Of the 167 MPS I births 118, 38 and 11 were classified as Hurler, Hurler-Scheie and Scheie respectively. The birth prevalence for MPS I across this period calculates to 1.07/100,000 births. The prevalence of the sub-syndromes of MPS I were Hurler syndrome: 0.76/100,000, Hurler-Sheie syndrome 0.24/100,000 and Sheie syndrome 0.07/100,000.

Table 1 lists the birth prevalence by syndrome and compares our results with others reported in the literature [8-15]. For overall prevalence of MPS I, there is good agreement (within ± 5%) between our results and those reported from studies that examined reasonably large populations except for substantially lower rates observed for Germany which the authors termed exploratory[15].

**Table 1:** Prevalence studies of MPS I

**Study *Region***	**Ascertainment period**	**No. of cases**	**No. of live births**	**Syndrome subtype**	**Birth Prevalence/100,000**
Lowry (1971) [8]*British Columbia*	1952 – 1968	7	606,157	All	1.15
				Hurler	0.99
				Scheie	0.16
Lowry (1990) [9]*British Columbia*	1952 – 1986	7	665,702	All	0.77
				Hurler	0.69
				Scheie	0.08
Nelson (1997) [10]*N. Ireland*	1958 – 1985	14	839,520	All	1.66
				Hurler	1.30
				Hurler-Scheie	0.36
				Scheie	No cases
Nelson (2003) [11]*Western Australia*	1969 – 1996	6	641,178	All	0.93
				Hurler	0.93
				Hurler-Scheie	No cases
				Scheie	No cases
Poorthuis (1999) [12]*Netherlands*	1970 – 1996	82	6,871,909	All	1.19
Meikle (1999) [13]*Australia*	1980 – 1996	38^†^	3,344,000	All	1.14
Hutchesson (1998) [14]*West Midlands UK*	1981 – 1991	7	707,720	All	0.99
Baehner (2005) [15]*Germany*	1980 – 1995	93	13,410,924	All	0.69
				Hurler	0.61
				Hurler-Scheie	0.03
				Scheie	0.05
This Study *England & Wales*	1981 – 2003	167	15,611,220	All	1.07
				Hurler	0.76
				Hurler-Scheie	0.24
				Scheie	0.07

† 10 prenatal diagnoses were additionally observed.

### Survival

**Figure 2:**
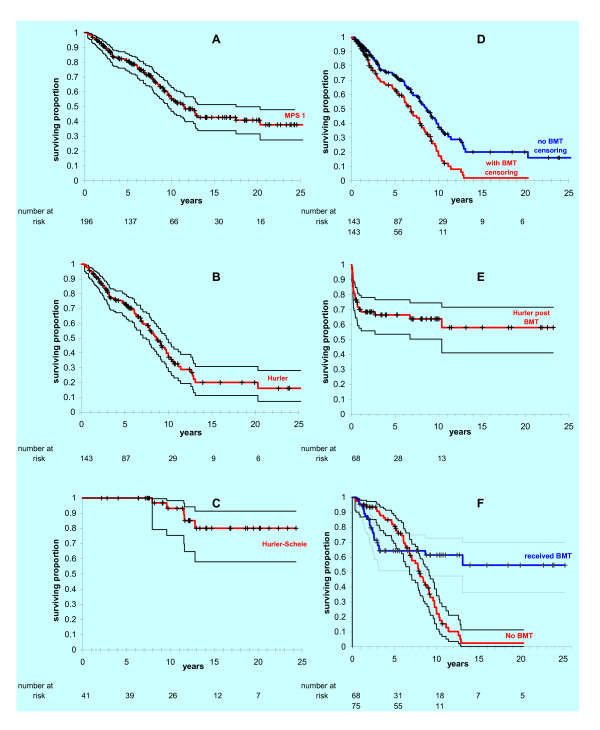
**Kaplan Meier survival curves**. A: All MPS I patients' survival from birth; B: Hurler patients' survival from birth; C: Hurler-Scheie patients' survival from birth; D: Hurler patients' survival from birth with and without censoring for bone marrow transplant; E: Hurler patients' survival from time of bone marrow transplantation; F: Hurler patients' survival from birth for those receiving or not receiving bone marrow transplant. Approximate 95% confidence intervals are shown for curves A-C, E and F but are omitted from D to aid clarity.

Of the 196 MPS I patients 85 had died. The Kaplan-Meier survival curve and approximate 95% CI for all 196 MPS I patients is shown in Figure 2A. Median survival estimated from this plot was 11.6 years, 95% CI: 9.5 to 13.7 (see Table 2 which lists mean and median survival for each syndrome where these were calculable). The survival curve for the 143 Hurler patients is shown in Figure 2B. Median survival from this plot was 8.7 years, 95% CI: 7.6 to 9.7. It is clear that the curve for all MPS I patients is dominated by the 79 deaths among Hurler patients. The survival plot for 41 Hurler-Scheie patients is shown in Figure 2C. There was insufficient information for estimation of median survival but it is obvious this is superior to that for Hurler patients. Of the 12 Scheie patients one was deceased (aged 17.5 years) and the remaining 11 alive with ages ranging 14.1 to 24.6 years as of May 2005.

**Table 2:** Median and mean survival of MPS I patients

	**SURVIVAL (years)**	
	**median (approximate 95% CI)**	**mean (approximate 95% CI)**

MPS I (ALL)	11.6 (9.5 to 13.7)	14.6 (13.0 to16.1)
HURLER (ALL)	8.7 (7.6 to 9.7)	10.5 (8.8 to12.2)
HURLER with censoring for BMT	6.8 (5.6 to 8.0)	6.6 (5.7 to 8.4)
HURLER that received BMT	Can not estimate	15.6 (12.5 to 18.8)
HURLER that did not receive BMT	8.0 (7.0 to 8.9)	7.9 (7.0 to 8.8)
HURLER post-BMT survival	Can not estimate	14.4 (11.5 to 17.4)
HURLER-SCHEIE	Can not estimate	21.6 (19.3 to 24.0)
SCHEIE	Can not estimate	Can not estimate (1 death only)

Twenty nine patients (25 Hurler-Scheie and 4 Scheie) had received ERT but the date of commencement was unrecorded. Due to the small proportion receiving ERT and the short time that ERT has been available it was not possible to model survival of these patients post-treatment. The unlikelihood of a dramatic short-term effect on mortality amongst milder syndrome patients means it is very unlikely that the survival curves would be materially altered by not censoring these patients at the time they started ERT.

### Bone marrow transplant therapy and survival

Two Hurler-Scheie and 65 Hurler patients were in receipt of BMT. The effect of this on the survival curve for Hurler patients was examined by censoring those patients in receipt of BMT at the time they received BMT and comparing the resulting curve with that for Hurler patients irrespective of BMT therapy (i.e. without censoring for receipt of BMT (Figure 2D)). The curves are significantly different according to the log rank test (Hazard Ratio 0.58, chi squared p = 0.0004). The difference could be attributed to superior prognosis of those selected for BMT, to benefits upon survival of receiving BMT or to both.

Survival of Hurler patients who received BMT after receipt of BMT is shown in Figure 2E. The mean age at transplant was 1.33 years. A particularly hazardous period is the year immediately following transplant. This might be attributed to poor short-term prognosis for those selected for transplant, or more likely inherent risks of the BMT procedure or perhaps relative frailty of Hurler patients compared with other patients in receipt of BMT. After one year post-BMT probability of survival remains good (68%, 66%, and 64% at 1, 5, and 10 years post-BMT). These results are similar to those reported in a recent pan-European study [16]. The survival from birth of Hurler patients who subsequently did and did not receive BMT is shown in Figure 2F.

## Discussion

In this retrospective study, utilising a registry of patient data encompassing most, if not all, UK patients with MPS I, the prevalence of the disease was 1.07/100,000. The prevalence not only falls within all definitions of an orphan disease but meets the definition of an ultra-orphan disease (less then 2/100,000) as defined by the National Institute for Health and Clinical Excellence [17]. This is the largest reported prevalence study, involving nearly twice as many cases and a slightly bigger population pool of the next largest (see Table 1). The prevalence obtained was similar to that from other studies with a large population pool from which cases were ascertained. All studies have been undertaken in developed countries with predominantly Caucasian populations. The prevalence of the sub-syndromes of MPS I reported here are of the same order of magnitude as other studies that have been able to measure the prevalence or have reported it (see Table 1) [15]. The prevalence of Hurler syndrome is highest and Sheie the lowest. The effect of detection bias due to the more ready identification of severe compared to less severe syndromes within these findings is unclear.

As expected survival analysis indicated the relative severity of Hurler's syndrome, with median survival of 8.7 years, and the relative mildness of Sheie syndrome relative to Hurler-Sheie syndrome and all MPS I patients. Median survival could not be calculated for the Hurler-Sheie or Sheie syndromes at present due to the longer duration of follow up required.

Although 60% (25/41) of Hurler-Scheie and 33% (4/12) of Sheie patients had received treatment with enzyme replacement therapy the small sample sizes and the too recent emergence of treatment precluded the possibility of estimating an effect of the therapy on survival. With regard to bone marrow transplant in Hurler patients, censoring those patients who received a transplant reduced survival indicating the better prognosis of those selected to receive BMT and/or the benefit of BMT. The survival of Hurler patients post-BMT was very similar in this study to that recently reported for a pan-European multicentre study [16] which contained twice as many cases (n = 148 compared to 68), with survival rates at one year being 68% and remaining relatively stable for those followed for up to 10 years.

Surveillance of long term effectiveness and of adverse events relating to drug and other treatments is best reliant on large, population-based, disease-specific, well organised studies. For very rare conditions, disease specific registries would represent the best way of monitoring treatment effectiveness in terms of survival and other patient-centred outcome measures. Furthermore, they are very useful for measuring adverse events which are doubly difficult to monitor through hopefully low frequency of such events and the rarity of the disease. Whilst the treatment licensing bodies can request post-marketing studies, in addition to post-marketing reporting of adverse events, these usually only collect data on patients treated with the specific intervention and thus untreated patients or patients treated with other interventions (licensed or otherwise) are often not included [18]. Furthermore, the US Food and Drug Administration documentation demonstrates that post-marketing studies committed to by the pharmaceutical industry are infrequently implemented or completed [19]. The clinical registries of individual or multiple specialist centres set up for disease-specific conditions and unique charitable registries, such the one utilised in this report set up by the Society for Mucopolysaccharide Diseases UK [20], may offer the only reliable source of information on the natural history and the effect of therapies for specific rare conditions. With more therapies on the horizon, their likely marketing approval under orphan drug legislation and relative great expense, it is important that properly functioning registries be created or supported if they already exist, to record data on all patients whether treated with emerging technologies or not.

## Conclusion

This study, with the most extended follow up to date in a large target population, sets the birth prevalence of MPS I at 1.07/100,000 live births and median survival at 11.6 years.

## Competing interests

The authors declare that they have no competing interests.

## Authors' contributions

DM conceived and designed the study. MJC undertook statistical analyses and drafted the manuscript. EW provided clinical advice and input to all parts of the work and the manuscript. CL built and maintained the MPS I database.
